# Unusual B-Lymphoid Blastic Crisis as Initial Presentation of Chronic Myeloid Leukemia Imposes Diagnostic Challenges

**DOI:** 10.1155/2022/9785588

**Published:** 2022-06-25

**Authors:** Nouran Momen, Bora Baysal, Sheila Jani Sait, Joseph Tario, You-Wen Qian

**Affiliations:** ^1^Clinical & Chemical Pathology Department, Cairo University, Giza, Egypt; ^2^Department of Pathology, Roswell Park Cancer Institute, 665 Elm Street, Buffalo, NY 14203, USA

## Abstract

Chronic myeloid leukemia (CML) is a clonal hematopoietic stem cell disorder, characterized by reciprocal translocation *t*(9,22) (q34; q11), leading to increased myeloid proliferation. Most cases are diagnosed in the chronic phase (CP). However, a minority of cases can be present in the blastic phase (BP). In most patients with CML-BP, the blasts have a myeloid phenotype, however, in 20–30% of cases, the blasts have a lymphoid phenotype, mostly a B-cell phenotype. It is challenging to differentiate CML B-lymphoblastic phase (CML-BLP) from Ph + primary B-acute lymphoblastic leukemia (B-ALL) especially when the CML-BLP is the initial presentation of the disease, which is uncommon. We report here an unusual case of CML-BLP as an initial presentation of the disease without typical CML morphological findings. This case demonstrates diagnostic challenges and emphasizes the importance of an integrated approach using morphology, multiparametric flow cytometry, cytogenetic studies, and molecular studies to render an accurate diagnosis.

## 1. Introduction

Chronic myeloid leukemia (CML) is a myeloproliferative disorder characterized by the presence of the Philadelphia (Ph) chromosome generated by the reciprocal translocation *t*(9,22) (q34; q11), leading to the proliferation of granulocytes and their precursors. Around 50% of newly diagnosed CML patients are asymptomatic and are usually diagnosed when a white blood cell (WBC) count is found to be elevated during a routine medical examination [1]. Detection of BCR-ABL1 fusion is required for the diagnosis of CML. Meanwhile, Ph chromosome/BCR-ABL1 fusion is also the most common recurrent cytogenetic abnormality in B-lymphoblastic leukemia (B-ALL) in adults, representing around 25–30% of adult B-ALL cases [2]. Different fusion proteins are produced from different BCR-ABL1 fusion transcripts depending on the breakpoints within the breakpoint cluster region (BCR). The most common fusion proteins are p210 and p190, in which the breakpoints are located within the major BCR (M-BCR) and minor BCR (m-BCR), respectively. In CML, almost all cases are associated with the p210, and p190 is rarely detected, whereas in Ph + B-ALL, almost all cases are associated with p190, and p210 is detected in a minority of adult cases [3].

The natural progression of the disease usually follows a triphasic course; a chronic phase (CP), an accelerated phase (AP), and a blastic phase (BP). Most CML cases are diagnosed in the CP. Patients in AP have a blast count of 10–19% in the blood or bone marrow, and BP is characterized by more than 20% blasts in the blood or bone marrow. In most patients with CML-BP, the blasts show a myeloid phenotype, however, in 20–30% of cases the blasts show a lymphoid phenotype, usually B-lymphoblasts [4]. The BP can be the initial presentation of the disease in approximately 15% of adults and 5% of children, with blasts expressing a lymphoid phenotype in 20 to 30% of these cases [5], with rare *T* or mixed phenotypes [6]. In those cases, with AP or BP as the initial presentation with no prior history of CML, the presence of basophilia, myeloid hyperplasia, and micromegakaryocytes (“dwarf” megakaryocytes) may be suspicious of underlying CML [4].

Here, we describe a case which had none of the above findings and yet was diagnosed as a case of CML-BLP to discuss the challenges we faced to reach a final diagnosis.

## 2. Case Presentation

**Figure 1:**
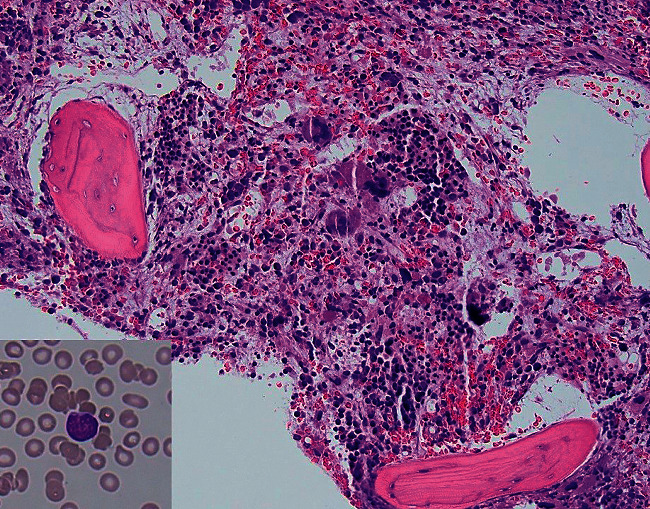
Hematoxylin and Eosin (H & E) stained bone marrow biopsy section showing hypercellular marrow with myeloid hypoplasia, erythroid hyperplasia, and increased atypical megakaryocytes (x200). An enclosed picture shows the morphology of the immature cells on a Wright-Giemsa-stained bone marrow aspirate smear (x400).

**Figure 2:**
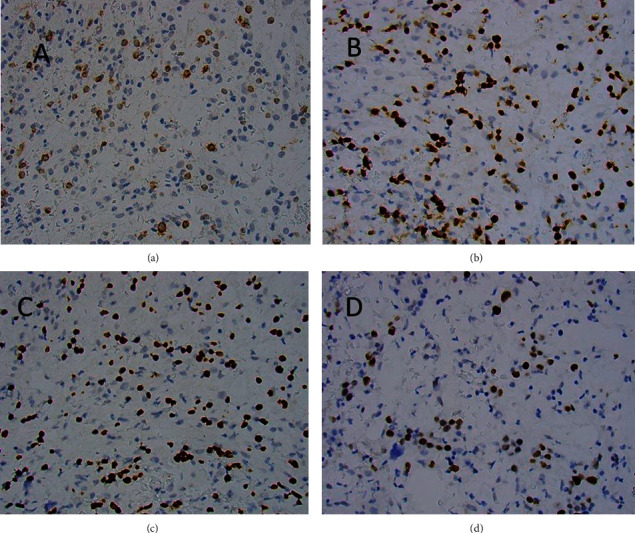
Immunohistochemistry-stained bone marrow biopsy sections showing: A-Positive CD34 staining in immature cells, B- Positive TdT staining in immature cells, C- Positive PAX-5 staining in immature cells, and D- Positive c-MYC staining in immature cells (x200).

We report a 69-year-old female with a history of atrial fibrillation and hyperlipidemia, presenting with a 1-month history of shortness of breath with an acute worsening of the condition. When she came to the emergency room (ER), she was hemodynamically stable and her oxygen saturation was 95% on room air. No organomegaly on the physical exam. Her complete blood count (CBC) showed pancytopenia with a white blood cell (WBC) of 1100/*μ*L, a hemoglobin (Hg) level of 4.5 g/dL, and a platelet count of 107,000/*μ*L. The differential count was within the normal ranges and no abnormal cells were seen on the blood smear. Her INR was 1.85 and her prothrombin time (PT) was 20.8 seconds. A bone marrow aspirate and biopsy (BMAB1) revealed about 6% immature cells on examination of the aspirate smears, while the biopsy examination showed a hypercellular bone marrow (80% cellularity) with a population of atypical immature B-cells (about 20–25%) together with myeloid hypoplasia, erythroid hyperplasia, atypical megakaryocytes, and marrow fibrosis (MF-2) as shown in Figure 1. These cells showed positive expression of CD34, TdT, CD20, PAX-5, and c-MYC by immunohistochemistry as seen in Figure 2.

**Figure 3:**
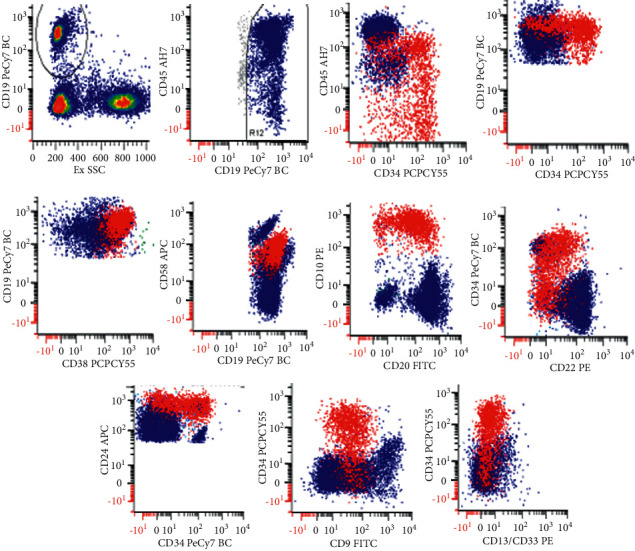
Flow cytometric immunophenotyping of the immature population in the bone marrow. The blasts express CD45 (heterogenous), CD19, CD34 (heterogeneous), CD38 (moderate), CD58 (bright), CD20, CD10, CD22 (dim), CD24 (heterogenous), CD9 (dim) and are negative for CD13 and CD33. B-lymphoblasts are red, mature B cells are blue.

Immunophenotyping by flow cytometry was performed on the bone marrow aspirate sample. A low-sensitivity leukemia panel was initially performed where a population of immature B-cells was identified. Although a hematogone population phenotype was considered in the differential, the moderate intensity of CD38 and homogenous expression of CD10 were concerning. A high-sensitivity COG B-precursor leukemia minimal residual disease (MRD) panel was subsequently performed, where 281,400 events were collected. B cells were measured at 10.7% of total cells and exhibited a spectrum of maturation. However, a subset of B cells expressed an atypical phenotype with heterogenous expression of CD45, CD34, and CD20 together with positive expression of CD19, CD10, CD38, CD9, CD24, bright expression of CD58, and dim expression of CD22. This population showed negative expression of CD13 and CD33. This immature population represented around 1.9% of total cells and was suspicious of a B-lymphoblastic leukemia/lymphoma (Figure 3).

**Figure 4:**
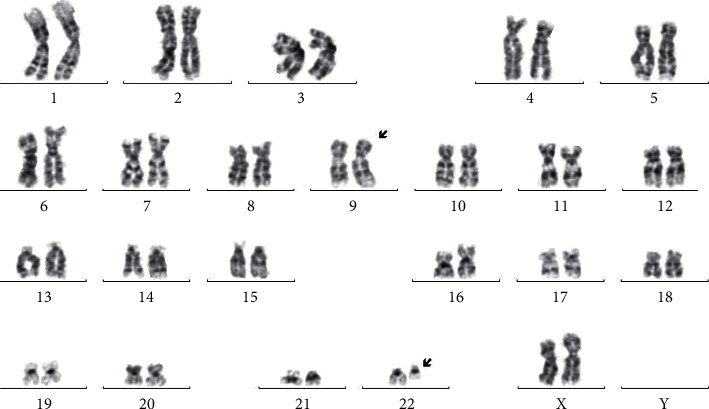
Conventional karyotyping shows positive *t*(9, 22).

Karyotyping performed on the bone marrow aspirate sample revealed a female karyotype with *t*(9,22) detected in 3/20 cells as illustrated in Figure 4.

**Figure 5:**
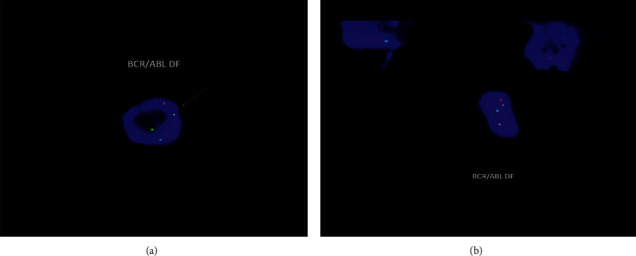
FISH showing positive BCR/ABL1 translocation. (a) Fusion signal in a cell with morphologic feature of blast. (b) Fusion signal in a cell with morphologic features of a band neutrophil.

Fluorescence in situ hybridization (FISH) studies were positive for BCR/ABL1 fusion seen in 24/200 cells examined. Among the BCR/ABL1 fusion positive cells, some have morphologic features of blasts, while cells with features of band neutrophils are also noted (Figure 5). and was negative for immunoglobulin heavy chain (IGH) gene rearrangements.

A subsequent bone marrow aspirate and biopsy (BMAB2) performed a few days later showed similar morphologic and immunophenotypic findings to those seen in BMAB1. Karyotyping detected *t*(9; 22) in 3/20 cells. PCR for BCR/ABL performed on the bone marrow aspirate sample was positive for BCR-ABL1 e13a2 (b2a2, p210) fusion transcript, detected at an International Scale (IS) of more than 55%. A bone marrow aspirate and biopsy performed after 2 weeks (BMAB3) also showed similar findings to those noted in BMAB2.

**Figure 6:**
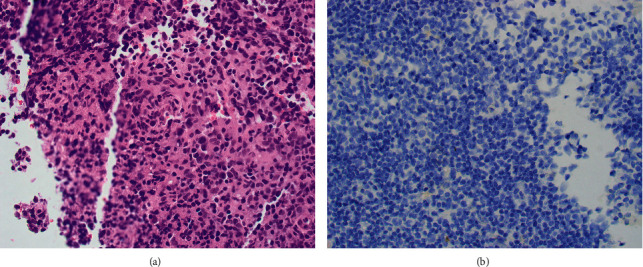
(a) H&E stained LN needle biopsy section showing a non-necrotizing granuloma with no evidence of lymphoid infiltrate (x200). (b) Immunohistochemically stained lymph node needle biopsy section showing negative staining for TdT (x200).

A PET/CT skull-base to mild-thigh was done after BMAB3 that showed an intense hypermetabolic left axillary lymph node (LN) measuring 1.5 × 1 cm, while the spleen was normal in size with no metabolic signature of a tumor. A needle biopsy was taken from the left axillary LN and revealed a non-necrotizing granuloma with no evidence of lymphoid infiltrate (Figure 6). FISH studies done on the LN touch imprints were positive for BCR/ABL1 fusion in 22/200 cells.

Two months later, the patient came for a follow-up. Examination of the blood smear showed mildly left-shifted neutrophilia with 2% blasts. Bone marrow aspirate and biopsy (BMAB4) at this time revealed a hypercellular marrow (70%) with myeloid hyperplasia, 5% blasts, reduced erythroid precursors, and mild fibrosis (MF-1). Immunostains on the BM biopsy for CD34, TdT, and PAX-5 were positive in around 5% of immature B-cells. Immunophenotyping on the bone marrow aspirate revealed an abnormal population comprising 2.4% of total cells with positive expression of CD45 (dim), CD19, HLADR, CD7 (dim), CD13, CD34, CD38, CD10 (subset), CD33 (heterogenous), CD15 (dim subset), CD123 (dim), CD71 (heterogenous), CD117 (dim subset), TdT, cCD22 (dim) and cCD79a (heterogenous). This population is consistent with abnormal B-lymphoblasts with aberrant myeloid antigen expression. Karyotyping detected *t*(9; 22) in 11/20 cells. In addition, deletion 7 was detected in 8/20 cells. FISH studies were positive for BCR/ABL1 fusion in 162/200 cells and deletion 7 was detected in 52/200 cells analyzed. The emergence of deletion 7 by karyotyping and FISH as a new cytogenetic abnormality, along with the abnormal population detected morphologically and phenotypically, was highly suggestive of a case of CML in an accelerated phase.

Therapy was initiated with daily Dasatinib. Follow-up CBC around two months later showed normocytic normochromic anemia and leukopenia. Bone marrow examination (BMAB5) revealed a mildly hypercellular bone marrow for age (50%) with progressive trilineage hematopoiesis and 1% blasts. A flow cytometric evaluation using the COG MRD panel, where 542,700 events were collected, showed no evidence of relapsed/residual disease. B cells represented around 2% of total cells and exhibited a spectrum of differentiation. FISH studies were positive for BCR/ABL1 fusion in 5/200 cells and for deletion 7 in 3/200 cells analyzed. PCR analysis revealed an evident reduction in BCR-ABL1 e13a2 (b2a2, p210) fusion transcript (detected at an IS of 1.25). The patient has been clinically stable for about 6 months on therapy and has been evaluated for transplant. All five bone marrow findings are summarized in Table 1.

## 3. Discussion

### 3.1. Differentiation between CML-BLP and de Novo B-ALL

Although CML remains an indolent illness in the majority of patients, the blastic phase still represents a serious and often life-threatening complication requiring aggressive measures to control the disease. Differentiation between Ph + B-ALL and CML-LBP is very important since it has a significant impact on the patient's management and outcome. CML patients presenting with BP often have a much worse prognosis. Patients with Ph + B-ALL are usually treated with frontline regimens for B-ALL and tyrosine kinase inhibitors (TKI). Patients with CML-LBP are usually treated with TKI combined with intensive chemotherapy followed by allo-SCT in qualified patients [7].

Our patient was diagnosed with a CML-BLP rather than Ph + B-ALL for several reasons. First, the p210 BCR-ABL1 fusion was detected instead of the p190 BCR-ABL1. A diagnosis of CML-BLP should always be considered when the transcripts are p210 [5]. Fusion gene p190 can be detected in less than 1% of CML cases and is often associated with monocytosis in CML-CP [7]. In B-ALL, Ph chromosome/BCR-ABL1 fusion is the most common recurrent cytogenetic abnormality in adults. In adults with B-ALL, p190 is detected in around 60–70% of cases, and p210 is detected in around 30–40% of cases [8]. Second, the discordance between the percentage of blasts and the size of the Ph + clone is supportive of a diagnosis of CML-BLP. In CML-BLP, BCR/ABL1 is detected in both lymphoblasts and myeloid cells since the myeloid cells are part of the proliferative process. Therefore, the Ph + clone count is often higher than the blast count. Whereas in de novo Ph + B-ALL, BCR/ABL1 is restricted to the lymphoblasts thus the Ph + clone size is often close to blast count. In our case, all four bone marrow evaluations showed a higher Ph + clone count compared to the blast count in the corresponding bone marrow aspirate morphological evaluations. For example, BMAB4 had 5% blasts yet karyotyping detected *t*(9; 22) in 11/20 cells (55%), and the FISH studies were positive for BCR/ABL1 fusion in 162/200 cells analyzed (81%). Moreover, the *t*(9,22) was detected in the LN biopsy using FISH in the absence of immature cell infiltrate. Even further, BCR/ABL1 fusion is also seen in cells with band form nuclear features. Third, the patient had atypical megakaryocytic morphology and marrow fibrosis in BMAB1 and neutrophilia with left-shift in BMAB4, which are suggestive of an underlying myeloid neoplasm. CML-BP tends to have high WBC, neutrophilia, and immature myeloid cells in the blood [5]. Although the diagnosis of CML-AP was made on BMAB4, retrospectively, the increased B-lymphoblasts to 20–25% in BMAB1 represents a CML-BLP. The blast count varied between 2% and 25% in the four bone marrow samples examined over a period of two months. This case demonstrated that the B-lymphoblast count in CML AP or BLP may not be stable or even decrease without therapy. NCCN guideline version 3.2022 [9] also states that an increase in lymphoblasts is concerning and should be considered as a sign of an impending transformation to frank BP.

In our case, the bone marrow initially showed myeloid hypoplasia and erythroid hyperplasia. It is difficult to explain these findings in a CML case. The megakaryocytic series did not show the typical “dwarf” morphology expected to be seen in cases of CML. According to the 2018 WHO classification, megakaryocytes in CML may be normal or slightly decreased in number, but 40–50% of the cases exhibit moderate to marked megakaryocytic proliferation. In the chronic phase, megakaryocytes are usually smaller than normal, have hyposegmented nuclei, and are referred to as “dwarf” megakaryocytes but they are not true micromegakaryocytes, such as those seen in cases of the myelodysplastic syndrome [4].

### 3.2. Differentiation between B-Lymphoblasts and Hematogones

The other diagnostic challenge is to differentiate B lymphoblasts from hematogones (HGs). HGs may exhibit morphological characteristics indistinguishable from lymphoblasts. They may constitute 5% to more than 50% of the bone marrow differential count. It is especially difficult to distinguish minimal B-lymphoblastic involvement in bone marrow from HGs. It has been shown that B-lymphoblasts usually show overexpression of CD123, CD58, CD10, CD22, CD34, and underexpression of CD38, CD81, and CD45 compared to HGs. They may also show aberrant expression of other lineage markers. CD58 and CD81 are two important markers that can be used to improve the distinction between HGs and precursor B lymphoblasts. It was recommended that CD58 and CD81 should be included in flow cytometry panels done at the time of diagnosis, thus allowing reliable and rapid MRD monitoring, especially for patients with no aberrant markers on their lymphoblasts [10]. Another useful marker is CD38. It is brightly expressed on HGs throughout all stages of maturation until it is downregulated in mature B cells. Approximately 75% of B-ALL cases express CD38, with a dimmer intensity than that expressed on HGs. The applicability of the underexpression of CD38 in B-ALL as a discriminating marker has been demonstrated in various studies examining its utility in MRD testing [11].

Interestingly, in a study by Vrotsos et al., they reported detecting a small (less than 0.5%) abnormal B-lymphoblast population in cases of chronic phase CML in a significant minority of diagnostic bone marrow samples. After follow-up of patients, it was concluded that this finding does not inevitably herald progression to a B-lymphoblastic blast phase [12].

In our case, the initial low-sensitivity leukemia panel showed decreased CD38 intensity and homogenous CD10 expression, which was concerning. The subsequent COG MRD study detected a population of B-lymphoblasts with a bright expression of CD58 and a positive expression of CD9. Interestingly, the B-lymphoblastic population showed an antigenic shift after two months with aberrant expression of the myeloid antigen CD13. Our case also emphasizes the importance of CD38 intensity in differentiating B-lymphoblasts from HGs, especially when MRD evaluation is not available. CML-BLP with expression of antigens from more than 1 lineage is common and additionally expression of 1 or more myeloid-related antigens is frequently seen [4]. Nevertheless, the flow cytometry immunophenotypic profile was lacking the typical features of Ph + B-lymphoblasts where bright CD10 together with aberrant expression of CD13 and CD33 and loss of CD45 were frequently seen. CD117 and CD15 were positively expressed on the lymphoblasts in our case, which is not typical for cases of Ph + B-ALL.

### 3.3. C-MYC Expression on CML-BLP

In our case, the immature B cells showed positive expression of c-MYC by immunohistochemistry on BMAB1. Deregulated MYC is found in about half of human tumors, being more prevalent in hematological neoplasms. Approximately 2%–5% of cases of ALL show MYC rearrangements. A number of B-ALL cases, while not having MYC gene abnormalities, show high MYC expression [13]. It was also found that BCR-ABL upregulates MYC expression, which cooperates with BCR–ABL in the transformation from the chronic phase to the blastic phase in CML cases. Consistently, imatinib and other BCR–ABL inhibitors provoke downregulation of MYC [14]. It was found that MYC mRNA levels were elevated in cases of CML-BP and in CML-CP compared to healthy bone marrow samples and that high MYC expression levels correlate with poorer response to imatinib and progression to the blastic crisis [15]. While c-MYC may not help in differentiating between CML-BLP and Ph + B-ALL, it would be interesting to follow-up with the patient and assess MYC expression in the bone marrow.

### 3.4. Cytogenetic Changes in CML-BLP

At diagnosis, approximately 5% to 10% of cases of CML will show chromosomal abnormalities in addition to *t*(9; 22); however, additional chromosomal anomalies (ACAs) are identified in around 50% to 80% of CML-AP as the disease progresses. ACAs are also commonly acquired during the course of CML treatment. In fact, the acquisition of specific additional chromosomal abnormalities can be used as a criterion for advancement from CML-CP to CML-AP. The chromosomal changes that define a transformation to CML-AP include a second Philadelphia chromosome, isochromosome 17q, trisomy 8, trisomy 19, abnormalities of 3q 26.2, or a complex karyotype [16]. Some studies have shown that B-ALL with BCR-ABL1 is associated with a greater number of additional chromosomal changes when compared with CML in BLP [17]. Additionally, gains in chromosome 9 are associated with B-ALL with BCR-ABL1, whereas deletions in chromosome 9p are more commonly seen in CML in BLP. This chromosomal abnormality is postulated to play an important role in CML-BLP because 9p is home to genes important in B-cell differentiation (PAX5 and CDKN1A). Lymphoid BP has also been associated with deletion of p16/CDKN2A and numerical gains and breakpoints involving chromosomes 1q and 7p. However, neither of these changes can be used to definitively distinguish between CML-BLP and B-ALL [18].

Our patient developed Del 7 around two months after the first presentation. It was reported that monosomy 7 in CML is rare, with only a few reported cases [19]. It was also reported that in adult ALL cases, the presence of monosomy 7, as a sole secondary abnormality, was associated with a poor prognosis and shorter survival [20]. However, the emergence of del (7) in our case does not seem to correlate with disease progression. The role of del (7) in CML disease progression may need further observation.

Detecting cytogenetic abnormalities plays an important role in the diagnosis of hematologic malignancies. It is worthy to point out that in rare instances, some of these abnormalities may be constitutional and are not necessarily linked to the neoplastic process, leading to a diagnostic dilemma. For instance, very few cases have been reported to have a constitutional balanced *t*(9; 22) (q34; q11.2) cytogenetically mimicking the acquired *t*(9; 22) (q34; q11.2), characteristic of CML. These findings prove the necessity of a detailed investigation of such cases together with correlation with all clinical findings to avoid an erroneous diagnosis of myeloid malignancies in the presence of rare constitutional cytogenetic abnormalities [21].

## 4. Conclusion

CML presenting with a B-lymphoblastic crisis as an initial presentation is very uncommon, and it may present without typical CML morphology. It is important to differentiate such cases from primary Ph + B-ALL due to the impact it has on the patient's management and prognosis. PCR testing, together with cytogenetic studies and multiparametric flow cytometry, serves as tools that help in reaching an accurate diagnosis. However, the importance of morphological evaluation and applying a comprehensive immunohistochemistry panel cannot be undermined. The prognosis of patients with CML-BP is poor and, without proper treatment, patients usually die. However, with TKI therapy, survival has markedly improved.

## Figures and Tables

**Table 1:** The bone marrow aspirate and biopsy findings during the disease course.

Bone marrow	Date	Morphology on aspirate	IHC on biopsy	Flow cytometry	Karyotyping	FISH	Molecular by PCR
BMAB1	6-3-21	6% immature cells on aspirate	20–25% immature B-cells on biopsy	1.9% B-lymphoblasts by COG MRD	*t*(9 : 22) in 1/20 cells	BCR/ABL124/200 cells	Not done
BMAB2	6-7-21	10% blasts	About 10% blasts	No COG MRD	*t*(9 : 22) in 3/20 cells	Not done	Positive for p210, IS of more than 55%.
BMAB3	6-24-21	2% blasts	2%	0.2% B-lymphoblasts by COG MRD	*t*(9 : 22) in 9/20 cells	BCR/ABL1 22/200 cells	Not done
BMAB4	8-16-21	5% blasts	5% B-lymphoblasts	2% B-lymphoblasts by COG MRD	46, XX, *t*(9; 22) [4/10] 45, XX, -7, *t*(9; 22) [6/10]	BCR/ABL1 162/200 cells	Not done
BMAB5	10-14-21	1% blasts	1% blasts	Negative COG MRD	46, XX [15]	BCR/ABL1 5/200 cells	Positive for p21, IS of 1.25%.

## Data Availability

Data available on request.
